# Microsatellite-Based Genetic Structure and Hybrid Detection in Alpacas Bred in Poland

**DOI:** 10.3390/ani11082193

**Published:** 2021-07-23

**Authors:** Angelika Podbielska, Katarzyna Piórkowska, Tomasz Szmatoła

**Affiliations:** 1 Department of Animal Molecular Biology, National Research Institute of Animal Production, Krakowska 1, 32-083 Balice, Poland; katarzyna.piorkowska@iz.edu.pl (K.P.); tomasz.szmatola@iz.edu.pl (T.S.); 2 Center for Experimental and Innovative Medicine, University of Agriculture in Krakow, Rędzina 1c, 30-248 Kraków, Poland

**Keywords:** alpacas, hybrids, microsatellite markers, population structure, genetic diversity

## Abstract

**Simple Summary:**

Alpacas (*Vicugna pacos*) are South American members of the tribe Lamini of the Camelidae family. They are bred for their fiber, which is considered a luxury material. Interest in alpaca breeding is increasing in Poland, but the local alpaca population is relatively young and heterogeneous. The poor quality of alpaca fiber results from uncontrolled crossing with llamas (*Lama glama*). Hybridization between the two species is a well-known phenomenon among alpaca breeders worldwide and is the cause of poor fiber quality, which leads to economic losses. Microsatellite markers can distinguish alpacas from llamas and indicate the level of admixture. However, it is difficult to determine in which generation the admixture took place. The high genetic diversity of alpacas bred in Poland has emerged as a consequence of their mixed origins. In this context, the microsatellite markers recommended by the International Society for Animal Genetics have been shown to be highly useful for individual identification and parentage testing of alpacas.

**Abstract:**

This study aimed to characterize the population structure and genetic diversity of alpacas maintained in Poland using 17 microsatellite markers recommended by the International Society for Animal Genetics. The classification of llamas, alpacas, and hybrids of both based on phenotype is often difficult due to long-term admixture. Our results showed that microsatellite markers can distinguish alpacas from llamas and provide information about the level of admixture of one species in another. Alpacas admixed with llamas constituted 8.8% of the tested individuals, with the first-generation hybrid displaying only 7.4% of llama admixture. The results showed that Poland hosts a high alpaca genetic diversity as a consequence of their mixed origin. More than 200 different alleles were identified and the average observed heterozygosity and expected heterozygosity values were 0.745 and 0.768, respectively, the average coefficient of inbreeding was 0.034, and the average polymorphism information content value was 0.741. The probability of exclusion for one parent was estimated at 0.99995 and for two parents at 0.99999.

## 1. Introduction

The alpaca (*Vicugna pacos*) belongs to the South American Camelid (SAC) group and the tribe Lamini of family Camelidae, together with the llama (*Lama glama*), another widely domesticated species. In contrast, the vicuna (*Vicugna vicugna*) and guanaco (*Lama guanicoe*) are wild representatives of this family. These animals naturally inhabit the Andes, stretching across Peru, Chile, Bolivia and Argentina [1]. In South America, llamas are bred for transport, meat and wool, while alpacas are bred mainly for fiber and wool [2].

In Poland, alpaca breeding began in 2004 and the interest in this subject is constantly increasing. Due to their meagre ecological requirements, these animals have adapted well to the Polish climate. Their breeding requires limited human involvement compared to other domesticated animals and can be carried out in unspecialized farm buildings with readily available feed, which is why breeding in Poland is becoming competitive [3].

Alpacas were first kept for didactic and recreational purposes in agritourist farms [4,5]. However, they are now bred primarily for their fiber, which is regarded as a luxury material [6]. The estimated wool production is 10 tons per year [7]. Washed alpaca wool costs EUR 35–50 kg^−1^, and the price of one alpaca is approximately EUR 1400–1800. Despite the high price of the animals and their products, there is a great demand for this raw material due to its quality and properties [8].

Alpaca fiber quality can be negatively affected by hybridization and uncontrolled crossing of alpacas with llamas, which has occurred since the Spanish conquest of South America [2]. Unfortunately, due to this long-term hybridization, their recognition based on phenotype is often ineffective [9]. Moreover, all SACs have the same number of chromosomes (2*n* = 74) and can cross either naturally or through artificial insemination, producing fertile offspring, which can be backcrossed both with llama and alpaca [10].

Comparative analysis among different species using a microsatellite marker based on Bayesian clustering has been used to analyze interspecies admixture. Alpaca hybridization with other SACs was proven in previous studies [11,12]. Additionally, the levels of dog (*Canis familiaris*) and wolf (*Canis lupus* ssp.) introgression were determined in a similar manner [13,14,15,16,17,18] as well as pig (*Sus domestica*) and wild boar (*S. scrofa*) [19], different species of zebra (*Equus* sp.) [20], crocodile (*Crocodylus* sp.) [21], hare (*Lepus* sp.) [22] and red-legged partridge (*Alectoris* sp.) [23]. The aim of this study was to assess the genetic diversity of alpacas and determine whether it is possible to identify alpaca–llama hybrids kept in Poland on the basis of microsatellite markers, as it is assumed that their exclusion from breeding programs could contribute to the improvement of fiber quality.

## 2. Materials and Methods

### 2.1. Sample Collection and Microsatellite Genotyping

Hair follicle and buccal swabs of 234 animals were collected from 5 farms in The Alpaca and Llama Breeding Society, 3 farms in The Polish Alpaca Breeders Association and from Cracow and Wroclaw zoos. The sample consisted of 216 alpacas, 15 llamas, 1 control llama–alpaca hybrid and 2 putative hybrids (indicated as such by the breeders themselves). Fifteen llama samples were collected as a control group. The list of tested animals is included in the Supplementary Materials in Table S1, while the genotypes of the tested animals are shown in Table S2.

DNA was extracted with the Sherlock AX Kit (A&A Biotechnology, Gdynia, Poland) following the suggested manufacturer protocol. DNA concentration and quality were assessed using a MaestroNano device (Maestrogen, Las Vegas, NV, USA). 

In this study, 17 microsatellite markers recommended by the International Society for Animal Genetics (ISAG) were employed. Two multiplex PCR reactions were prepared and optimized for amplification. The first reaction included 12 markers and the second included 5 markers (Table 1). Both the multiplex PCRs were performed using SimplyAmp Thermo Cycler (Applied Biosystems, Foster City, CA, USA).

The reaction mixture contained 11.2 μL Type-it Microsatellite PCR Kit (QIAGEN GmbH, Hilden, Germany), 1.2 μL primer mix and 1 μL DNA (30 ng/μL). PCR conditions for all reactions consisted of an initial denaturation of 95 °C for 5 min, followed by 28 cycles of 95 °C for 30 s, 60 °C for 90 s and 72 °C for 30 s, with a final extension step of 60 °C for 30 min. Capillary electrophoresis was performed using a 3130xl Genetic Analyser (Applied Biosystems, Foster City, CA, USA). Each reaction well contained 11 μL formamide, 0.4 μL GeneScan™ 500 LIZ™ dye Size Standard (Applied Biosystems, Foster City, CA, USA) and 1 μL of PCR product. Samples were denatured for 5 min at 95 °C. The electrophoresis results were analyzed using GeneMapper v. 4.0 (Applied Biosystems, Foster City, CA, USA).

### 2.2. Population Structure Analysis

The population structure and admixture of our sample was investigated using the Bayesian approach, implemented in STRUCTURE 2.3.4 [29]. Four different analyses were carried out. For the first, 234 individuals (alpacas, llamas and putative hybrids) were treated as if they belonged to a unique population. In the second, we differentiated the 15 llamas from the rest of the individuals. Both analyses were performed with a burn-in period of 100,000 and 200,000 iterations and *K* ranging from 1 to 4 with 10 runs for each *K*. In the third analysis, we assigned individuals to one of three groups: 216 alpacas, 15 llamas and 3 putative hybrids. This analysis was performed with a burn-in period of 100,000 and 200,000 iterations fitting *K* from 1 to 6 with 10 runs for each *K*. 

STRUCTURE HARVESTER [30] was used to select the best *K* in all following stages and visualize it with CLUMPAK [31]. Pure-bred alpacas and llamas were considered individuals with the estimated membership coefficient value of *q* ≥ 0.98 [11]. We used the ClumpIndFile.output file from the third analysis (standard output of CLUMPAK, where the values were averaged) for *K* = 2 to analyze the *q* value.

We carried out an additional STRUCTURE analysis (fourth), where we used 15 llamas and the same number of pure-bred alpacas. We selected 15 alpacas for which *q* ≥ 0.98 in the previous analysis in STRUCTURE. This analysis was aimed at checking whether the unequal amount of sample had an influence on the final results of the obtained admixture. The analysis was performed similarly to the three previous analyses, with *K* ranging from 1 to 4. 

### 2.3. Statistical Analysis

Genetic diversity indices were computed for the 216 alpacas. The number of alleles (Na), observed heterozygosity (Ho), expected heterozygosity (He), coefficient of inbreeding (Fis), heterozygote deficit (*p*-value), Hardy–Weinberg equilibrium (HWE) and genetic distance among all samples were calculated using GenAlEx 6.5 [32,33]. Allelic richness was obtained by the HP-RARE 1.0 [34,35]. The polymorphism information content (PIC), null allele frequencies (Fnull), non-exclusion probability (first parent: NE-1P), non-exclusion probability (second parent: NE-2P), non-exclusion probability (identity): NE-I and combined non-exclusion probability for the first, second parent and identity (CNE-1P, CNE-2P, CNE-I) were calculated in CERVUS 3.0.7 [36]. Genetic distance dendrogram was constructed using the unweighted pair group method with arithmetic mean (UPGMA) [37]. The tree was generated by iTOL v5 [38].

In addition, private alleles of llama, alpaca and shared alleles were identified using GENEPOP 4.7 [39,40] in two independent analyses. All collected specimens were used for the first analysis, except 3 suspected hybrids. For the second analysis, only individuals with *q* ≥ 0.98 were used.

## 3. Results

### 3.1. Genetic Structure

**Figure 1:**
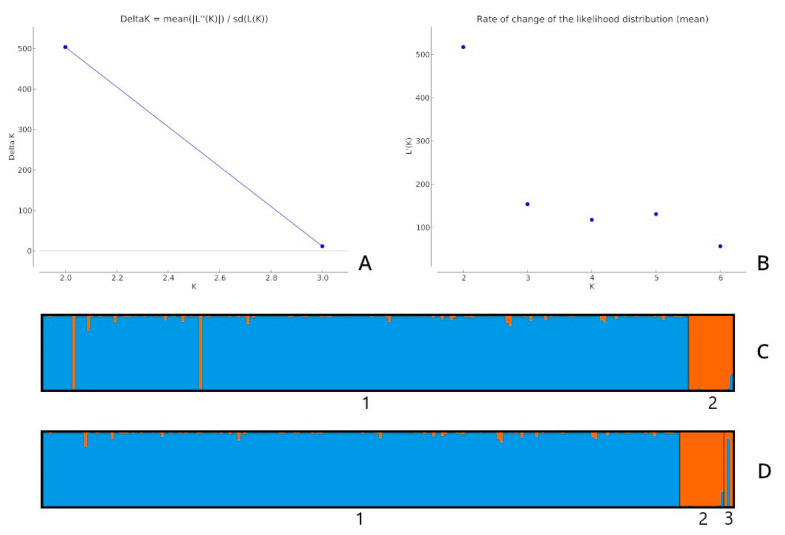
(**A**) Delta *K* values obtained with STRUCTURE HARVESTER for the second analysis (llamas and the rest of the individuals); (**B**) rate of change in the likelihood distribution (mean) for the third analysis (individuals divided into three populations: alpacas, llamas and putative hybrids); (**C**) structural analysis for *K* = 2 obtained for the second analysis; (**D**) structural analysis for *K* = 2 obtained for the third analysis. The number 3 indicates potential hybrids, 2 corresponds to the population of llamas, and 1 represents the remaining individuals.

For all four analyses in STRUCTURE, the best *K* was *K* = 2 (Figure 1A,B).

The ten runs delivered an identical score of 0.999 (all ten runs for *K* = 2 presented very similar results), as shown in Figure 1C,D. *q* for all analyses was similar. Figure 1C shows that two potential hybrids were found in the llama population.

The results of the fourth analysis did not differ significantly from the previous ones. For each alpaca, the *q* value remained at *q* ≥ 0.98. The last tested llama (as in previous analyses) indicated introgression with alpaca (data not shown).

**Figure 2:**
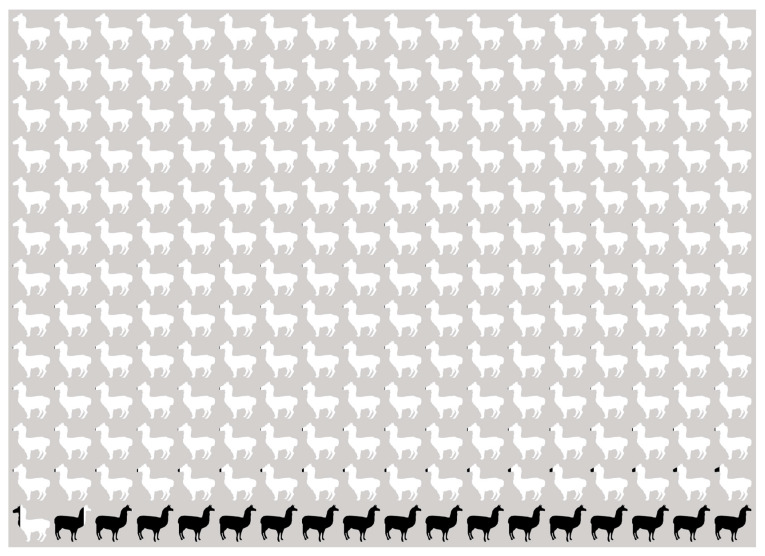
Percentage of individual membership in all tested animals, based on coefficient value (*q*). Alpaca: white color, llama: black color.

**Figure 3:**
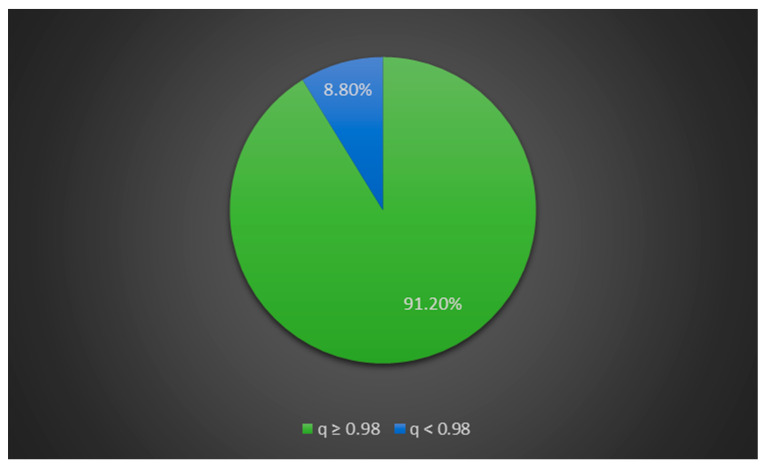
Percentage of pure-bred alpacas (*q* ≥ 0.98) and alpaca × llama hybrids (*q* < 0.98) across all tested alpacas.

The percentage of shared alpaca and llama membership across the 234 tested individuals is shown in Figure 2. Alpacas with llama admixture accounted for 8.8% of the entire alpaca dataset, while pure-bred alpacas accounted for 91.2% (Figure 3).

Based on the STRUCTURE analyses, it was found that the proposed microsatellite markers distinguished alpacas from llamas well. Among the studied individuals, various levels of admixture were observed. *q* values of the three putative hybrids are given in Table 2. 

Putative hybrid 1 was the daughter of a llama whose DNA profile analysis was also performed. Control hybrid 2 was the daughter of an alpaca mother and a llama father. The DNA profiles of its parents were analyzed. Its mother was classified as a pure-bred alpaca, while the father was a pure-bred llama. Control hybrid 2 had only 7.4% llama admixture. This could be related to the fact that the mother had numerous private alpaca alleles and the father had only shared, non-private llama alleles. The parents of potential hybrid 3 were not tested. The DNA profiles of these individuals are shown in Table 3.

The private alleles of alpacas and llamas and shared alleles are presented in Table 4. After removing alpaca–llama crosses (*q* < 0.98), some shared alleles were reclassified as private to alpacas or llamas.

Among the animals removed from the second analysis and showing llama admixture, six had private llama alleles. During this analysis, allele 182 of locus LCA5 disappeared (after the first analysis, it was only in alpacas). This allele was found in individuals indicated by STRUCTURE as alpacas admixed with llamas (*q* < 0.98). Thus, it can be assumed that this allele may be typical of llamas; however, the low number of llamas tested meant that we could not confirm this. Alleles 142 (LCA37), 230 (LCA66), 284 (LCA99) and 187 (LGU50) were shared in the first analysis and private to alpacas in the second; all of these alleles were found in the llama, admixed with alpaca.

Based on the presence of alleles private to llamas (Table 4), it can be concluded that putative hybrid 1 and putative hybrid 3 were in fact llamas. Putative hybrid 1 had allele 146 at locus LCA37, which was unidentified in alpacas, and potential hybrid 3 had alleles 255 at locus LCA8, 217 at locus LGU49, 191 at locus LGU50 and 136 at locus YWLL43-X (Table 3), which were not observed in the tested pure-bred alpacas.

### 3.2. Genetic Diversity

The population of alpacas maintained in Poland showed a high level of genetic diversity. A total of 201 different alleles were observed. The average number of alleles per locus was 11.8, ranging from 5 alleles in YWLL46 and LGU50 to 18 alleles in LCA66 (Table 5).

Allelic richness (Ar) ranged from 2.885 (YWLL46) to 8.037 (YWLL44), with an average of 6.14. Observed heterozygosity (Ho) ranged from 0.382 (YWLL46) to 0.871 (LCA37), with an average of 0.745. Expected heterozygosity (He) ranged from 0.406 (YWLL46) to 0.874 (YWLL44), with an average of 0.768. The coefficient of inbreeding (Fis) ranged from −0.088 (LCA5) to 0.384 (YWLL43-X), with an average of 0.034. Four of the 17 loci showed significant deviation from HWE: LCA19, LCA99, YWLL43-X and YWLL36. PIC ranged from 0.368 (YWLL46) to 0.862 (YWLL44) and the average PIC was 0.741. The non-exclusion probability when the parental genotype was known (NE-1P) ranged from 0.403 (YWLL44) to 0.916 (YWLL46), and the combined non-exclusion probability (CNE-1P) in this case was 4.997 × 10^−5^. This means that the combined exclusion probability when the genotype of one parent was known (CE-1P) was 0.99995. The non-exclusion probability when genotypes of both parents were known (NE-2P) ranged from 0.251 (YWLL44) to 0.788 (YWLL46), and the combined non-exclusion probability (CNE-2P) in this case was 1.0 × 10^−7^. This means that the combined exclusion probability when the genotypes of both parents was known (CE-2P) was 0.99999. The non-exclusion probability for identity of two unrelated individuals (NE-I) ranged from 0.028 (YWLL44) to 0.391 (YWLL46), and the combined non-exclusion probability for identity (CNE-I) in this case was 2.144 × 10^−20^. The frequency of null alleles (Fnull) ranged from −0.045 (LCA5) to 0.246 (YWLL43-X), with an average of 0.021.

**Figure 4:**
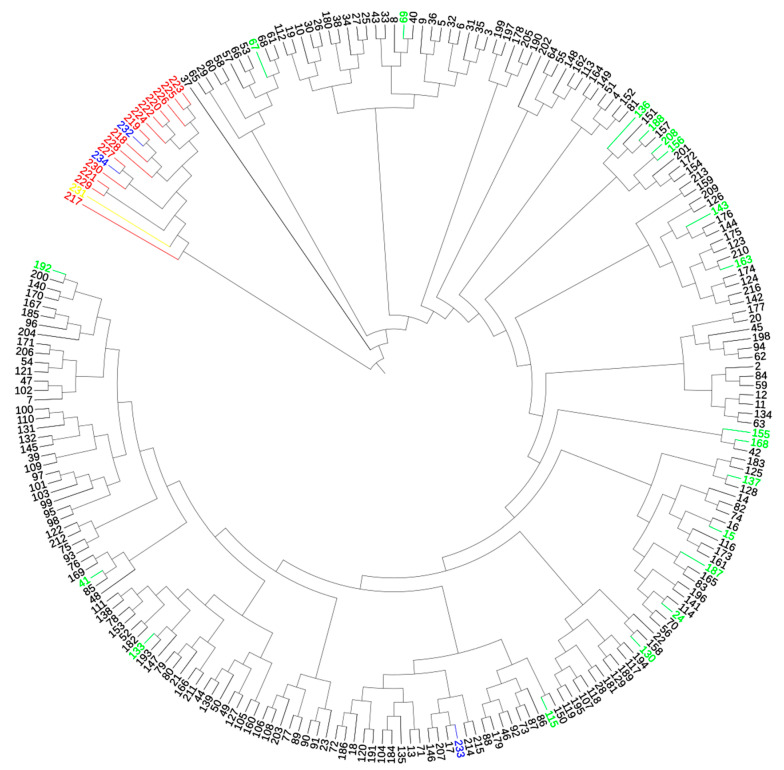
Dendrogram of genetic distance between all individuals by UPGMA algorithm. Black color—alpacas with *q* ≥ 0.98; red color—llamas with *q* ≥ 0.98; blue color—three putative hybrids; green color—alpacas with *q* < 0.98; yellow color—llama with *q* < 0.98.

The dendrogram of genetic distance showed two distinct clusters among all individuals (Figure 4). 

The results coincided with those obtained in the STRUCTURE program. The first main cluster consisted of llamas and two putative hybrids (Putative Hybrid 1 and Putative Hybrid 2). The second main cluster was divided into 20 subclusters. It consisted of pure-bred alpacas as well as those with an admixture of llamas and Control Hybrid 2. Alpacas with an admixture of llama were distributed in different subclusters.

## 4. Discussion

### 4.1. Population Structure and Llama–Alpaca Hybrids 

In this study, we aimed to distinguish alpacas from llamas and alpaca–llama hybrids using microsatellite markers and a Bayesian clustering approach. Additionally, the genetic diversity of alpacas bred in Poland was explored and the usefulness of this panel of markers for individual identification and parentage testing was assessed.

The population of alpacas maintained and bred in Poland is relatively young and heterogeneous since the animals were imported from various countries. Many individuals came from Chile because the local regulations regarding the export of animals are relatively lenient compared to neighboring countries. Unfortunately, not all imported animals had certificates of pedigree registration, which may explain why 8.8% of the studied population was admixed with llama. Nevertheless, the Polish Alpaca Breeders Association and Alpaca and Llama Breeding Society strive to organize the breeding of alpacas in Poland and the selection of animals to maintain herds with the most valuable traits.

To meet the expectations of alpaca breeders, on 23 January 2021, the “Act on the Organization of Breeding and the Reproduction of Farm Animals” (JOURNAL OF LAWS OF THE REPUBLIC OF POLAND, 2021) entered into force. Under the act, *V. pacos* is recognized as livestock in Poland. The classification of alpacas as farm animals is mainly associated with assessing their utility value, obtaining their genetic profile and selecting individuals for mating in proper breeding conditions. Additionally, Poland applies a lower value-added tax (VAT) for livestock animal service, including parentage testing research. This is why an attempt was made to identify alpaca–llama hybrids and eliminate the animals of suspected hybrid origin from herd books.

Hybridization between alpacas and llamas is a phenomenon known among breeders all over the world. Following the Spanish conquest in the 16th century, Andean native domestic livestock populations were reduced by 80–90% within the first 100 years of contact [2]. Traditional breeders call alpaca × llama hybrids “wari”. They are then classified as llama-wari or llama-like and paqowari or alpaca-like, depending on the phenotype. Other terms given to hybrids include wakayu, waritu, wayki [41] and huarizos [11], which also appear in the literature.

In this study, we observed that alpacas and llamas mostly cluster apart, but some hybrids were detected. Among the tested alpacas with *q* < 0.98, six possessed private llama alleles. Other non-pure alpacas displayed a llama admixture. When hybridization is occasional, the gene flow between species may only transfer a negligible portion of the genome. With more frequent hybridization, alleles that flow from donor to recipient species may represent segregated variability. This phenomenon may impact gene flow if the alleles underlying a specific genetic variant are transferred non-randomly from donor to recipient species [42].

Hybridization between different South American Camelids species has been proven before [9,43], and it has been found to occur more frequently in domestic than wild populations [44]. Previous studies based on microsatellite markers in Bolivian alpacas also found that many individuals exhibit a llama admixture in their genome and indicated that the two species, despite genetic selection, have not split [11].

Since microsatellite markers are inherited according to Mendel’s laws, we can use them to determine the admixture of one species in another. However, using this method, it is difficult to determine which generation this admixture occurred in. “Admixture alleles” can be inherited from generation to generation by randomly segregating alleles to descendants. In this study, the first-generation hybrid had 7.4% llama admixture, with the mother being a pure-bred alpaca and the father a pure-bred llama. Based on an assignment using Bayesian methods, first-generation hybrids should have a *q* value of 0.5. These individuals should be intermediate between the two clusters in the two-population model [45]. Smaller values may suggest mixed populations. It must be remembered that alpacas and llamas have not yet been genetically separated. Unfortunately, the Spanish conquest of South America irreversibly destroyed the original genetic diversity of the SACs. 

The alpaca–llama crosses revealed by the population structure analysis must have obtained “admixture alleles” several generations ago, as Polish breeders do not allow hybridization due to the risk of reducing the quality of the fiber, which results in breeding and economic losses. In the Andes, alpacas were specifically crossed with llamas for 25 years at the turn of the 20th and 21st centuries. Male alpacas were mated with female llamas to increase the population of animals producing more expensive “alpaca fiber”. However, male llamas have been crossed with female alpacas to increase fleece weight and income [46].

The llama with an admixture of alpaca was identified in this study by four alleles found in alpacas, and the level of admixture was 20.7%. This proves that the level of admixture increases with the number of inherited private alleles. According to some authors [47], genotyping of markers that carry private alleles can be a valuable tool for distinguishing between these two species. However, further studies on a larger population are required.

Another problem of hybridization is determining the origin of alpacas—that is, whether its ancestor was a vicuna, llama or guanaco. Kadwell et al. [9] showed that the ancestor of the alpaca is the vicuna (*V. vicugna*), while the ancestor of the llama is the guanaco (*L. guanicoe*). These authors suggested changing the name from *Lama pacos* to *V. pacos*. However, according to Barreta et al. [43], the estimated pairwise distances between alpacas and llamas are shorter than between alpacas and vicunas. In this case, further research on alpacas and their origin seems necessary. If the ancestor of the alpaca is the vicuna, which is famous for the unusual properties of its fiber, hybridization with llamas would be an unfavorable phenomenon.

### 4.2. Genetic Diversity

The obtained results revealed a high level of genetic variability among alpacas bred in Poland. Most of the markers were highly polymorphic. In diversity studies, the utility of markers designates more than four alleles per loci [48]. In the present study, the least polymorphic loci were YWLL46 and LGU50, with five alleles, but they were classified as applicable. In a study by Paredes et al. [49], who analyzed over 20 STR loci, five alleles were found in the least polymorphic marker.

The allelic richness value of 2.885 was obtained for YWLL46, which indicated its poor utility. However, the values that we obtained, except for the YWLL46, were higher than those obtained by other authors for otters (*Lutra lutra*) [50], hares (*Lepus europaeus*) [51], arapaimas (*Arapaima gigas*) [52] and the black soldier fly (*Hermetia illucens*) [53], while they were lower than reported for dolphins (*Stenella coeruleoalba*) [54].

In a previous study, Paredes et al. [55] reported that for measuring genetic variation, the average heterozygosity should range from 0.3 to 0.8. In the present study, lower values were observed for YWLL43-X and YWLL46; therefore, it may be necessary to substitute the markers used with others. Polish alpacas showed an average Ho of 0.745 (0.382–0.853) and an average He of 0.768 (0.406–0.874), so they fell within the required range and displayed even higher results than those obtained by other authors [48,49,55,56,57]. At nine loci, a lower Ho was observed in comparison to He, which may indicate a heterozygous deficit in the studied populations, suggesting a need for a more conscious crossing of alpacas in Poland in the future, aimed at increasing the diversity of males for mating. However, some authors [57,58] found that the observed heterozygosity was always lower than expected.

In the present study, the average Fis value in the alpaca population was 0.034, so it can be said that no unfavorable inbreeding phenomenon was observed among alpacas kept in Poland. A lower Fis was recorded in Peru [55] and Bolivia [56], although higher values were observed in the former in other studies [49,57,58].

Four of the seventeen tested markers showed a significant deviation from HWE. Other studies also revealed deviations in the HWE, with 13 of 22 markers [56], 12 of 69 markers [55] and 8 of 15 markers [58] showing deviations. These aberrations may result from selective mating, population substructure, sample shortage, low polymorphism and selection of homozygotes, which may also reduce heterozygosity [55]. The most likely cause of significant deviations from HWE in the four locations of the studied individuals may be the use of the same males in herds for mating females.

In turn, the PIC parameter supported the usefulness of this marker panel for genetic analyses. Moreover, a PIC of >0.5 for a microsatellite marker shows high polymorphic content. In the present study, the average PIC was 0.741. Similar results were obtained in the other studies [55,59,60].

A potential cause of null microsatellite alleles is poor primer annealing due to nucleotide sequence divergence through point mutations or indels in one or both of the flanking primers, differential amplification of alleles of different sizes or failure of PCR due to poor template quality [61]. The Wahlund effect or inbreeding can result in heterozygous deficits relative to the Hardy–Weinberg equilibrium that may be misinterpreted as evidence for the existence of zero alleles. Nevertheless, it must be assumed that null alleles are locus-specific [61]. When the null allele frequency is greater than 0.2, the marker should be removed from the parentage analysis. In the present study, the YWLL43-X locus showed low null allele frequency (0.2461). However, this could be an error, and the associated heterozygote deficit may be due to a sex bias since this marker is linked to the X-sexual chromosome. Nevertheless, our results showed that the frequency of null alleles is related to the heterozygote deficit.

The values of NE-1P, NE-2P and NE-I indicated YWLL46 as the least and YWLL44 as the most useful marker. Nevertheless, the tested markers proved to be more helpful than those used for parentage analysis in the wild boar (*S. scrofa*) [62] and goat (*Capra hircus*) [63] populations. However, the evaluation of the microsatellite markers used for the pedigree analysis in plateau pika (*Ochotona curzoniae*) [64] and giant grouper (*Epinephelus lanceolatus*) [65] showed better values than those obtained by us. This is further proof that the YWLL46 should be removed, because this marker underestimates the values in every analysis.

In the present study, the CPE1 was 0.99995 and CPE2 0.99999, which is higher than that obtained for alpacas [59], llamas and guanacos [66]. In the latest studies on animals bred in Poland, namely horses (*Equus caballus*) [67], pigs (*S. domestica*) [68] and dogs (*C. familiaris*) [69], lower values were also obtained. The results obtained for alpacas illustrate the utility of the tested markers for parentage testing.

## 5. Conclusions

This study was the first research on the structure of the population and genetic diversity of alpacas bred in Poland. However, it should be noted that this population is relatively young. Nevertheless, these preliminary studies can significantly impact the development of breeding strategies in the future. Based on the analysis of microsatellite markers, we have shown that it is possible to distinguish alpacas from llamas and estimate the level of admixture in the genomes of both species. However, the identification of hybrids should still be verified using mtDNA, Y chromosome or other markers, such as SNP, and on a higher number of individuals.

## Figures and Tables

**Table 1:** Characteristic of 17 microsatellite loci included in the first and second multiplex PCR reactions.

Locus	Primer (5′-3′) Forward	Primer (5′-3′) Reverse	Concentration F + R (μM) *	Dye	Size Range (bp)	Multiplex	Reference
LCA5	GTGGTTTTTGCCCAAGCTC	ACCTCCAGTCTGGGGATTTC	3	VIC	178–218	1	[24]
LCA8	GCTGAACCACAATGCAAAGA	AATGCAGATGTGCCTCAGTT	2.5	NED	211–261		[24]
LCA19	TAAGTCCAGCCCCACACTCA	GGTGAAGGGGCTTGATCTTC	2.5	6—FAM	80–122		[24]
LCA37	AAACCTAATTACCTCCCCCA	CCATGTAGTTGCAGGACACG	3	PET	124–174		[24]
LCA56	ATGGTGTTTACAGGGCGTTG	GCATTACTGAAAAGCCCAGG	2.5	NED	130–168		[25]
LCA65	TTTTTCCCCTGTGGTTGAAT	AACTCAGCTGTTGTCAGGGG	2.5	6—FAM	159–193		[25]
LCA66	GTGCAGCGTCCAAATAGTCA	CCAGCATCGTCCAGTATTCA	2.5	6—FAM	216–266		[25]
LCA94	GTCCATTCATCCAGCACAGG	ACATTTGGCAATCTCTGGAGAA	2.5	PET	187–213		[26]
LCA99	CAGGTATCAGGAGACGGGCT	AGCATTTATCAAGGAACACCAGC	2.5	VIC	263–297		[26]
LGU49	TCTAGGTCCATCCCTGTTGC	GTGCTGGAATAGTGCCCAGT	2.5	PET	219–255		[27]
LGU50	CTGCTGTGCTTGTCACCCTA	AGCACCACATGCCTCTAAGT	2.5	NED	183–201		[27]
YWLL44	CTCAACAATGCTAGACCTTGG	GAGAACACAGGCTGGTGAATA	4	VIC	84–136		[28]
YWLL29	GAAGGCAGGAGAAAAGGTAG	CAGAGGCTTAATAACTTGCAG	2.5	6—FAM	210–236	2	[28]
YWLL36	AGTCTTGGTGTGGTGGTAGAA	TGCCAGGATACTGACAGTGAT	2.5	PET	142–180		[28]
YWLL40	CACATGACCATGTCCCCTTAT	CCAGTGACAGTGTGACTAAGA	2.5	6—FAM	174–200		[28]
YWLL43-X	ATACCTCTCTTGCTCTCTCTC	CCTCTACAACCATGTTAGCCA	2.5	VIC	124–156		[28]
YWLL46	AAGCAGAGTGATTTAACCGTG	GGATGACTAAGACTGCTCTGA	2	NED	84–109		[28]

* Concentration of forward and reverse primer in primer mixture. All microsatellites were dinucleotide.

**Table 2:** *q* values of putative alpaca–llama hybrids.

	Putative Hybrid 1		Control Hybrid 2		Putative Hybrid 3	
	alpaca	llama	alpaca	llama	alpaca	llama
*q*	0.002	0.998	0.926	0.074	0.003	0.997

**Table 3:** DNA profiles of the hybrid and putative hybrids. ^a^, alleles found only in llamas; ^b^, alleles found only in alpacas.

Locus	Potential Hybrid 1	Control-Hybrid 2	Potential Hybrid 3
LCA5	188/190	188/202 ^b^	194/
LCA8	245/	239/241	241/255 ^a^
LCA19	86/	86/102 ^b^	86/
LCA37	144/146 ^a^	140/156 ^b^	132 ^b^/150
LCA56	139/	139/141	137/139
LCA65	169/173	171/	169/175
LCA66	220/260	224/226 ^b^	224/
LCA94	193/	191/199 ^b^	191/193
LCA99	278/286	282/288 ^b^	286/
LGU49	231/239	221 ^b^/231	217 ^a^/239
LGU50	193/	187 ^b^/193	191 ^a^/193
YWLL44	96/	86/122 ^b^	96/112
YWLL29	220/	218/220	218/220
YWLL36	156/	150/156	152/154
YWLL40	186/	186/188	186/
YWLL43-X	158/	152 ^b^/156	136 ^a^/
YWLL46	105/109	97/105	103/

**Table 4:** Alleles private to alpacas and llamas and shared alleles across the 17 microsatellite loci. Alleles with ^a^ were reclassified in two independent analyzes in the GENEPOP.

	All Individuals except Three Putative Hybrids			Individuals with *q* ≥ 0.98		
Locus	Alleles Identified Only in Alpacas	Alleles Identified Only in Llamas	Alleles Shared between Both Species	Alleles Identified Only in Alpacas	Alleles Identified Only in Llamas	Alleles Common to Both Species
LCA5	182 ^a^, 186, 192, 196, 200, 202, 204, 206	-	188, 190, 194	186, 192, 196, 200, 202, 204, 206	-	188, 190, 194
LCA8	231, 251, 261	257	237, 239, 241, 243, 245, 249, 253 ^a^, 255 ^a^	231, 251, 253 ^a^, 261	255 ^a^, 257	237, 239, 241, 243, 245, 249
LCA19	88, 96, 98, 100, 102, 104, 106, 108, 112, 116, 120	-	86, 90, 92 ^a^, 94	88, 92 ^a^, 96, 98, 100, 102, 104, 106, 108, 112, 116, 120	-	86, 90, 94
LCA37	132, 152, 154, 156, 158, 160, 164, 166, 168, 172	146, 184	134, 136, 140, 142 ^a^, 144, 148, 150	132, 142 ^a^, 152, 154, 156, 158, 160, 164, 166, 168, 172	146, 184	134, 136, 140, 144, 148, 150
LCA56	135, 143, 145, 149, 155, 161, 163, 165, 167	-	133, 137, 139, 141	135, 143, 145, 149, 155, 161, 163, 165, 167	-	133, 137, 139, 141
LCA65	165, 167, 177, 179, 181, 183, 185, 187, 189, 191	-	169, 171, 173, 175	165, 167, 177, 179, 181, 183, 185, 187, 189, 191	-	169, 171, 173, 175
LCA66	226, 229, 231, 232, 236, 238, 240, 242, 246, 256, 262	-	220, 222, 224, 228, 230 ^a^, 254, 260	226, 229, 230 ^a^, 231, 232, 236, 238, 240, 242, 246, 256, 262	-	220, 222, 224, 228, 254, 260
LCA94	195, 199, 201, 205, 207	-	189, 191, 193	195, 199, 201, 205, 207	-	189, 191, 193
LCA99	264, 272, 276, 280, 288, 292	-	268, 274, 278, 282, 284 ^a^, 286, 290, 294	264, 272, 276, 280, 284 ^a^, 288, 292	-	268, 274, 278, 282, 286, 290, 294
LGU49	221, 229, 233, 235, 237, 245, 247	217	225, 227 ^a^, 231, 239, 241, 243	221, 229, 233, 235, 237, 245, 247	217, 227 ^a^	225, 231, 239, 241, 243
LGU50	183, 189	191	187 ^a^, 193, 195	183, 187 ^a^, 189	191	193, 195
YWLL44	94, 108, 110, 116, 118, 120, 122, 124, 128	-	86, 96, 98, 102, 104, 106, 112, 114	94, 108, 110, 116, 118, 120, 122, 124, 128	-	86, 96, 98, 102, 104, 106, 112, 114
YWLL29	214, 222, 224	232	216 ^a^, 218, 220, 226, 228	214, 216 ^a^, 222, 224	232	218, 220, 226, 228
YWLL36	142, 162, 164, 168, 172, 174, 176, 178	-	148, 150, 152, 154, 156, 158, 170	142, 162, 164, 168, 172, 174, 176, 178	-	148, 150, 152, 154, 156, 158, 170
YWLL40	180, 184, 190	-	182, 186, 188	180, 184, 190	-	182, 186, 188
YWLL43-X	130, 140, 146, 152, 160	142	136 ^a^, 144, 148, 150, 156, 158	130, 140, 146, 152, 160	136 ^a^, 142	144, 148, 150, 156, 158
YWLL46	113	107	97, 103, 105, 109	113	107	97, 103, 105, 109

**Table 5:** Genetic diversity indices across 17 microsatellite markers used in this study.

Locus	Na	Ar	Ho	He	Fis	*p* Value	HWE	PIC	NE-1P	NE-2P	NE-I	F (Null)
LCA19	15	5.652	0.682	0.680	−0.002	0.000	***	0.654	0.703	0.516	0.128	−0.0020
LCA65	14	7.672	0.853	0.866	0.015	0.811	ns	0.852	0.424	0.267	0.032	0.0077
LCA66	18	7.426	0.770	0.817	0.058	0.873	ns	0.801	0.508	0.336	0.050	0.0341
YWLL44	17	8.037	0.834	0.874	0.045	0.998	ns	0.862	0.403	0.251	0.028	0.0237
LCA5	11	5.336	0.843	0.775	−0.088	0.977	ns	0.742	0.610	0.431	0.084	−0.0454
LCA99	14	7.068	0.829	0.839	0.012	0.000	***	0.821	0.482	0.315	0.044	0.0090
LCA56	13	6.568	0.802	0.803	0.001	0.996	ns	0.779	0.547	0.372	0.063	0.0012
LGU50	5	4.044	0.700	0.679	−0.031	0.853	ns	0.625	0.739	0.573	0.157	−0.0176
LCA8	11	7.047	0.853	0.853	0.000	0.624	ns	0.836	0.458	0.294	0.039	−0.0012
LCA37	17	7.950	0.871	0.857	−0.017	0.621	ns	0.842	0.437	0.279	0.035	−0.0099
LCA94	8	5.831	0.816	0.813	−0.004	0.549	ns	0.787	0.547	0.371	0.061	−0.0023
LGU49	13	7.330	0.848	0.844	−0.005	0.900	ns	0.828	0.468	0.303	0.041	−0.0045
YWLL40	6	3.846	0.604	0.674	0.105	0.105	ns	0.615	0.750	0.588	0.165	0.0590
YWLL29	8	5.600	0.760	0.752	−0.011	0.638	ns	0.723	0.630	0.447	0.091	−0.0038
YWLL43-X	11	4.381	0.406	0.658	0.384	0.000	***	0.603	0.751	0.588	0.172	0.2461
YWLL46	5	2.885	0.382	0.406	0.057	0.972	ns	0.368	0.916	0.788	0.391	0.0359
YWLL36	15	7.776	0.820	0.870	0.057	0.014	*	0.857	0.415	0.260	0.030	0.0295
TOTAL	201											
AVERAGE		6.140	0.745	0.768	0.034			0.741	0.576	0.411	0.095	0.021
	**CNE-1P**		0.00004997		**CE-1P**		0.99995003			**CNE-I**	2.144 × 10^−20^	
	**CNE-2P**		0.00000010		**CE-2P**		0.9999999					

Key: ns = not significant, * *p* < 0.05, *** *p* < 0.001; Na, the number of alleles per locus; Ar, allelic richness; Ho, observed heterozygosity; He, expected heterozygosity; Fis, coefficient of inbreeding; *p* value, a deficit of heterozygotes; HWE, Hardy–Weinberg equilibrium; PIC, polymorphism information content; NE-1P, non-exclusion probability (first parent); NE-2P, non-exclusion probability (second parent); NE-I, non-exclusion probability (identity); F (null), frequency of null alleles; CNE-1P, combined non-exclusion probability (first parent); CNE-2P, combined non-exclusion probability (second parent); CE-1P, combined exclusion probability (first parent); CE-2P, combined exclusion probability (second parent); CNE-I, combined non-exclusion probability (identity).

## Data Availability

Not applicable.

## Supplementary Materials

The following are available online at https://www.mdpi.com/article/10.3390/ani11082193/s1, Table S1: List of tested animals with estimated membership coefficient value (*q*), Table S2: The genotypes of the tested animals.
