# Supplementary material for: Microsatellite-Based Genetic Structure and Hybrid Detection in Alpacas Bred in Poland
Source: Animals (Basel). 2021 Jul 23;11(8):2193. doi: 10.3390/ani11082193 (PMC8388510; doi:10.3390/ani11082193)
Supplement: Supplementary file 1 [file animals-11-02193-s001.zip › Table S1.pdf]

Table S1. List of tested animals with estimated membership coefficient value (q).

Sample	Takson	Origin of the sample	Estimated membership coefficient value (q)	
			Alpaca	Llama
1	*Vicugna pacos*	England	0.996	0.004
2	*Vicugna pacos*	England	0.997	0.003
3	*Vicugna pacos*	England	0.983	0.017
4	*Vicugna pacos*	Poland	0.996	0.004
5	*Vicugna pacos*	Poland	0.997	0.003
6	*Vicugna pacos*	Poland	0.996	0.004
7	*Vicugna pacos*	England	0.996	0.004
8	*Vicugna pacos*	England	0.994	0.006
9	*Vicugna pacos*	The Netherlands	0.997	0.003
10	*Vicugna pacos*	Poland	0.997	0.003
11	*Vicugna pacos*	Poland	0.995	0.005
12	*Vicugna pacos*	Chile	0.996	0.004
13	*Vicugna pacos*	Poland	0.996	0.004
14	*Vicugna pacos*	Poland	0.994	0.006
15	*Vicugna pacos*	Chile	0.801	0.199
16	*Vicugna pacos*	Poland	0.989	0.011
17	*Vicugna pacos*	Unknown*	0.996	0.004
18	*Vicugna pacos*	Chile	0.996	0.004
19	*Vicugna pacos*	Chile	0.984	0.016
20	*Vicugna pacos*	Poland	0.998	0.002
21	*Vicugna pacos*	Poland	0.994	0.006
22	*Vicugna pacos*	England	0.996	0.004
23	*Vicugna pacos*	England	0.995	0.005
24	*Vicugna pacos*	England	0.910	0.090
25	*Vicugna pacos*	Poland	0.996	0.004
26	*Vicugna pacos*	Belgium	0.994	0.006
27	*Vicugna pacos*	Belgium	0.988	0.012
28	*Vicugna pacos*	Belgium	0.989	0.012
29	*Vicugna pacos*	England	0.988	0.012
30	*Vicugna pacos*	Poland	0.994	0.006
31	*Vicugna pacos*	England	0.990	0.010
32	*Vicugna pacos*	Poland	0.998	0.002
33	*Vicugna pacos*	England	0.996	0.004
34	*Vicugna pacos*	Poland	0.986	0.014
35	*Vicugna pacos*	Poland	0.997	0.003
36	*Vicugna pacos*	Australia	0.998	0.002
37	*Vicugna pacos*	Belgium	0.995	0.006
38	*Vicugna pacos*	Belgium	0.991	0.009
39	*Vicugna pacos*	Unknown*	0.997	0.003
40	*Vicugna pacos*	Australia	0.992	0.008
41	*Vicugna pacos*	Poland	0.946	0.054

42	*Vicugna pacos*	England	0.995	0.005
43	*Vicugna pacos*	Poland	0.998	0.002
44	*Vicugna pacos*	Belgium	0.990	0.010
45	*Vicugna pacos*	Belgium	0.995	0.005
46	*Vicugna pacos*	England	0.997	0.003
47	*Vicugna pacos*	England	0.987	0.013
48	*Vicugna pacos*	Czech Republic	0.995	0.005
49	*Vicugna pacos*	Unknown*	0.986	0.014
50	*Vicugna pacos*	Unknown*	0.996	0.004
51	*Vicugna pacos*	Poland	0.993	0.007
52	*Vicugna pacos*	Unknown*	0.995	0.005
53	*Vicugna pacos*	Unknown*	0.990	0.010
54	*Vicugna pacos*	Unknown*	0.985	0.015
55	*Vicugna pacos*	Unknown*	0.995	0.005
56	*Vicugna pacos*	Unknown*	0.995	0.005
57	*Vicugna pacos*	Unknown*	0.994	0.006
58	*Vicugna pacos*	Unknown*	0.993	0.008
59	*Vicugna pacos*	Unknown*	0.993	0.007
60	*Vicugna pacos*	Unknown*	0.994	0.006
61	*Vicugna pacos*	Unknown*	0.988	0.012
62	*Vicugna pacos*	Unknown*	0.995	0.005
63	*Vicugna pacos*	Unknown*	0.993	0.007
64	*Vicugna pacos*	Unknown*	0.981	0.019
65	*Vicugna pacos*	Unknown*	0.995	0.006
66	*Vicugna pacos*	Unknown*	0.991	0.009
67	*Vicugna pacos*	Unknown*	0.894	0.106
68	*Vicugna pacos*	Unknown*	0.995	0.005
69	*Vicugna pacos*	Poland	0.976	0.024
70	*Vicugna pacos*	Poland	0.998	0.002
71	*Vicugna pacos*	Poland	0.997	0.003
72	*Vicugna pacos*	Poland	0.997	0.003
73	*Vicugna pacos*	Poland	0.995	0.005
74	*Vicugna pacos*	Poland	0.998	0.002
75	*Vicugna pacos*	Poland	0.996	0.004
76	*Vicugna pacos*	Poland	0.995	0.005
77	*Vicugna pacos*	Switzerland	0.996	0.004
78	*Vicugna pacos*	Poland	0.997	0.003
79	*Vicugna pacos*	Poland	0.995	0.005
80	*Vicugna pacos*	Poland	0.994	0.007
81	*Vicugna pacos*	Poland	0.989	0.011
82	*Vicugna pacos*	The Netherlands	0.984	0.016
83	*Vicugna pacos*	Chile	0.997	0.004
84	*Vicugna pacos*	USA	0.996	0.004
85	*Vicugna pacos*	Poland	0.998	0.002
86	*Vicugna pacos*	Poland	0.986	0.014
87	*Vicugna pacos*	Germany	0.984	0.017

88	*Vicugna pacos*	Poland	0.997	0.003
89	*Vicugna pacos*	Poland	0.995	0.005
90	*Vicugna pacos*	Poland	0.994	0.006
91	*Vicugna pacos*	Germany	0.997	0.003
92	*Vicugna pacos*	Poland	0.982	0.018
93	*Vicugna pacos*	Poland	0.998	0.002
94	*Vicugna pacos*	Poland	0.990	0.010
95	*Vicugna pacos*	Poland	0.988	0.012
96	*Vicugna pacos*	Poland	0.994	0.006
97	*Vicugna pacos*	Poland	0.996	0.004
98	*Vicugna pacos*	Poland	0.996	0.004
99	*Vicugna pacos*	England	0.995	0.005
100	*Vicugna pacos*	Poland	0.996	0.004
101	*Vicugna pacos*	Poland	0.992	0.008
102	*Vicugna pacos*	England	0.997	0.003
103	*Vicugna pacos*	Poland	0.998	0.002
104	*Vicugna pacos*	Unknown*	0.996	0.004
105	*Vicugna pacos*	Poland	0.996	0.004
106	*Vicugna pacos*	Unknown*	0.997	0.003
107	*Vicugna pacos*	Poland	0.997	0.003
108	*Vicugna pacos*	Unknown*	0.994	0.006
109	*Vicugna pacos*	Poland	0.989	0.011
110	*Vicugna pacos*	Unknown*	0.997	0.003
111	*Vicugna pacos*	Poland	0.998	0.002
112	*Vicugna pacos*	Unknown*	0.996	0.004
113	*Vicugna pacos*	Poland	0.992	0.008
114	*Vicugna pacos*	Unknown*	0.988	0.012
115	*Vicugna pacos*	Poland	0.922	0.078
116	*Vicugna pacos*	Unknown*	0.993	0.007
117	*Vicugna pacos*	Poland	0.997	0.003
118	*Vicugna pacos*	Unknown*	0.997	0.003
119	*Vicugna pacos*	Poland	0.996	0.004
120	*Vicugna pacos*	Unknown*	0.996	0.004
121	*Vicugna pacos*	Poland	0.994	0.006
122	*Vicugna pacos*	Unknown*	0.998	0.002
123	*Vicugna pacos*	Poland	0.997	0.003
124	*Vicugna pacos*	Unknown*	0.996	0.004
125	*Vicugna pacos*	Poland	0.996	0.004
126	*Vicugna pacos*	Unknown*	0.994	0.006
127	*Vicugna pacos*	Poland	0.992	0.008
128	*Vicugna pacos*	Unknown*	0.997	0.003
129	*Vicugna pacos*	Poland	0.996	0.004
130	*Vicugna pacos*	Unknown*	0.980	0.020
131	*Vicugna pacos*	Poland	0.995	0.005
132	*Vicugna pacos*	Unknown*	0.994	0.006
133	*Vicugna pacos*	Poland	0.949	0.051

134	*Vicugna pacos*	Unknown*	0.996	0.004
135	*Vicugna pacos*	Poland	0.995	0.005
136	*Vicugna pacos*	Unknown*	0.953	0.047
137	*Vicugna pacos*	Poland	0.974	0.026
138	*Vicugna pacos*	Unknown*	0.995	0.005
139	*Vicugna pacos*	Poland	0.996	0.004
140	*Vicugna pacos*	Unknown*	0.995	0.005
141	*Vicugna pacos*	Poland	0.988	0.012
142	*Vicugna pacos*	Unknown*	0.991	0.009
143	*Vicugna pacos*	Poland	0.974	0.026
144	*Vicugna pacos*	Unknown*	0.994	0.006
145	*Vicugna pacos*	Poland	0.996	0.004
146	*Vicugna pacos*	Unknown*	0.997	0.003
147	*Vicugna pacos*	Poland	0.997	0.003
148	*Vicugna pacos*	Unknown*	0.997	0.003
149	*Vicugna pacos*	Poland	0.992	0.008
150	*Vicugna pacos*	Unknown*	0.997	0.003
151	*Vicugna pacos*	Poland	0.997	0.003
152	*Vicugna pacos*	Unknown*	0.998	0.002
153	*Vicugna pacos*	Poland	0.998	0.002
154	*Vicugna pacos*	Unknown*	0.992	0.008
155	*Vicugna pacos*	Poland	0.910	0.090
156	*Vicugna pacos*	Unknown*	0.867	0.133
157	*Vicugna pacos*	Poland	0.995	0.005
158	*Vicugna pacos*	Unknown*	0.993	0.007
159	*Vicugna pacos*	Unknown*	0.992	0.008
160	*Vicugna pacos*	Unknown*	0.995	0.005
161	*Vicugna pacos*	Unknown*	0.987	0.013
162	*Vicugna pacos*	Unknown*	0.997	0.003
163	*Vicugna pacos*	Chile	0.938	0.063
164	*Vicugna pacos*	Unknown*	0.996	0.004
165	*Vicugna pacos*	Unknown*	0.993	0.007
166	*Vicugna pacos*	Unknown*	0.997	0.003
167	*Vicugna pacos*	Unknown*	0.995	0.005
168	*Vicugna pacos*	Unknown*	0.948	0.052
169	*Vicugna pacos*	Unknown*	0.998	0.002
170	*Vicugna pacos*	Unknown*	0.987	0.013
171	*Vicugna pacos*	Unknown*	0.996	0.004
172	*Vicugna pacos*	Unknown*	0.994	0.006
173	*Vicugna pacos*	Unknown*	0.996	0.004
174	*Vicugna pacos*	Unknown*	0.996	0.004
175	*Vicugna pacos*	Unknown*	0.997	0.003
176	*Vicugna pacos*	Unknown*	0.992	0.008
177	*Vicugna pacos*	Unknown*	0.984	0.016
178	*Vicugna pacos*	Unknown*	0.994	0.006
179	*Vicugna pacos*	Unknown*	0.997	0.003

180	*Vicugna pacos*	Unknown*	0.995	0.005
181	*Vicugna pacos*	Unknown*	0.993	0.007
182	*Vicugna pacos*	Unknown*	0.997	0.003
183	*Vicugna pacos*	Unknown*	0.995	0.005
184	*Vicugna pacos*	Unknown*	0.998	0.002
185	*Vicugna pacos*	Unknown*	0.998	0.002
186	*Vicugna pacos*	Unknown*	0.997	0.003
187	*Vicugna pacos*	Unknown*	0.946	0.054
188	*Vicugna pacos*	Unknown*	0.917	0.083
189	*Vicugna pacos*	Unknown*	0.994	0.006
190	*Vicugna pacos*	Unknown*	0.996	0.005
191	*Vicugna pacos*	Unknown*	0.997	0.003
192	*Vicugna pacos*	Unknown*	0.966	0.034
193	*Vicugna pacos*	Unknown*	0.998	0.002
194	*Vicugna pacos*	Unknown*	0.995	0.005
195	*Vicugna pacos*	Unknown*	0.991	0.009
196	*Vicugna pacos*	Unknown*	0.995	0.005
197	*Vicugna pacos*	Unknown*	0.994	0.006
198	*Vicugna pacos*	Unknown*	0.993	0.007
199	*Vicugna pacos*	Unknown*	0.983	0.017
200	*Vicugna pacos*	Unknown*	0.998	0.002
201	*Vicugna pacos*	Chile	0.996	0.004
202	*Vicugna pacos*	Unknown*	0.994	0.006
203	*Vicugna pacos*	Chile	0.992	0.008
204	*Vicugna pacos*	Unknown*	0.996	0.004
205	*Vicugna pacos*	Unknown*	0.985	0.015
206	*Vicugna pacos*	Unknown*	0.993	0.007
207	*Vicugna pacos*	Unknown*	0.986	0.014
208	*Vicugna pacos*	Unknown*	0.955	0.045
209	*Vicugna pacos*	Unknown*	0.992	0.008
210	*Vicugna pacos*	Unknown*	0.997	0.003
211	*Vicugna pacos*	Unknown*	0.998	0.002
212	*Vicugna pacos*	Unknown*	0.985	0.015
213	*Vicugna pacos*	Unknown*	0.986	0.014
214	*Vicugna pacos*	Unknown*	0.988	0.012
215	*Vicugna pacos*	Unknown*	0.993	0.007
216	*Vicugna pacos*	Unknown*	0.995	0.005
217	*Lama glama*	Unknown*	0.002	0.998
218	*Lama glama*	Unknown*	0.002	0.998
219	*Lama glama*	Unknown*	0.003	0.997
220	*Lama glama*	England	0.018	0.982
221	*Lama glama*	Hungary	0.004	0.996
222	*Lama glama*	Unknown*	0.002	0.998
223	*Lama glama*	Unknown*	0.002	0.998
224	*Lama glama*	Unknown*	0.002	0.998
225	*Lama glama*	Unknown*	0.003	0.997

226	*Lama glama*	Unknown*	0.005	0.995
227	*Lama glama*	Unknown*	0.002	0.998
228	*Lama glama*	Unknown*	0.002	0.998
229	*Lama glama*	Unknown*	0.006	0.994
230	*Lama glama*	Unknown*	0.018	0.982
231	*Lama glama*	Unknown*	0.207	0.794
232	*Lama glama*	Poland	0.002	0.998
233	*Vicugna pacos*	England	0.926	0.074
234	*Lama glama*	Unknown*	0.003	0.997

*It is believed that most individuals of unknown origin were imported from Chile.
